# The protective role of vitamin E against the effects of hyperthyroidism on the rat pituitary–ovary axis

**DOI:** 10.1007/s00418-025-02399-w

**Published:** 2025-07-03

**Authors:** Elfide Gizem Bakırhan, Süleyman Kaplan

**Affiliations:** 1 Department of Histology and Embryology, Faculty of Medicine, Adıyaman University, Adıyaman, Turkey; 2 Department of Histology and Embryology, Faculty of Medicine, Ondokuz Mayıs University, Samsun, Turkey

**Keywords:** Hyperthyroidism, Ovary, Pituitary, Rat, Vitamin E

## Abstract

This study investigated the effects of an experimentally induced hyperthyroidism model on the pituitary–ovarian axis and the possible protective role of vitamin E against these adverse effects. Wistar albino rats were divided into five groups of eight animals each: control (“Cont,” no any treatment); hyperthyroidism (“Hypert,” L-thyroxine at 0.3 mg/kg/day); “Sham” (1 ml/day corn oil); “Hypert + vit E” (L-thyroxine (0.3 mg/kg/day and 100 mg/kg/day/1 ml vitamin E); and “Vit E” (100 mg/kg/day/1 ml vitamin E) groups. At the end of the experiment, ovarian tissues were exposed to electron microscopic and stereological analyses. Thyroid stimulating hormone (TSH), free thyroxine (FT4), follicle stimulating hormone (FSH), luteinizing hormone (LH), and superoxide dismutase (SOD) levels, as well as catalase (CAT) enzyme activity, were also determined in blood serum samples. Ovarian follicle numbers and volumes; corpus luteum and cortex volume; body weights; and TSH, FSH, and SOD levels decreased significantly in the Hypert group compared with the Cont group. However, connective tissue volume, CAT enzyme activity, and FT4 levels increased in the Hypert group compared with the Cont group. Vitamin E supplementation was observed to play a protective role on antral follicle, corpus luteum, and connective tissue volumes, CAT activity, and SOD and FSH levels. Hyperthyroidism reduces the number of ovarian follicles and may cause infertility problems. The adverse effect of hyperthyroidism on the pituitary–ovarian axis can be ameliorated by means of vitamin E, since this may have a homeostatic effect on this axis, as shown by the biochemical, histopathological, and stereological analyses in this study.

## Introduction

Thyroid autoimmunity is the most common endocrine disorder, affecting 5–20% of women of reproductive age. The changes in the pituitary–ovarian axis caused by hyperthyroidism affect ovarian reserve, follicle development, and the structural integrity of the ovary. It is also thought to eventually lead to female infertility problems. However, it is still unclear whether these changes occur through the direct effect of thyroid hormones on the ovaries or on the pituitary and hypothalamus. The pituitary–thyroid axis and the hypothalamus–ovary axis are physiologically related and play a combined role as a single system in pathological conditions. According to experimental and clinical studies, the direct effects of thyroid hormones on the gonads are the result of the joint activity of stimulatory and inhibitory hormones released from the anterior pituitary (Hernandez 2018). The presence of thyroid hormone receptors in the ovary (oocytes, cumulus cells, and granulosa cells) and endometrial tissues, and the presence of thyroid antibodies in the follicular fluid, suggest that hyperthyroidism produces a direct cytotoxic reaction that damages the oocyte (Vissenberg et al. 2015). Examination of the effects of changes in thyroid hormone levels on the ovary during hyperthyroidism has revealed that the cells are mostly affected in terms of number or quality (Colicchia et al. 2014). A study investigating the effect of hyperthyroidism on follicle development in neonatal and non-adult rats reported a significant decrease in the total numbers of primordial, primary, and secondary follicles compared with a control group (Fedail et al. 2014).

Hyperthyroidism can affect hormone levels in both genders by causing changes in sex hormones and the proteins that carry these hormones in the blood. Vargas et al.’s hormonal evaluation determined an increase in follicle stimulating hormone (FSH) and luteinizing hormone (LH) levels in the postmenopausal age range and the presence of free hyperthyroxinemia with inappropriate normal TSH Vargas et al. 2017). However, there are also studies in literature showing normal FSH and LH levels in women with hyperthyroidism (Silva et al. 2018). Although the number of studies of hyperthyroidism is increasing, the limited amount of research into infertility caused by hyperthyroidism, especially in women, and the reporting of contradictory results, reveal the importance of studies on this subject.

Accelerated mitochondrial electron transport caused by the hormone-induced hypermetabolic state results in increased superoxide formation (Ramadan et al. 2021). Experimental studies and epidemiological data suggest that hyperthyroidism increases free radical production and lipid peroxide levels (Araujo et al. 2006). Excessive reactive oxygen species (ROS) production can lead to anovulation, dysfunctional oocytes, fertilization and implantation failure, and/or miscarriage (Wei et al. 2018). At the same time, factors such as oxidative stress, which cause disruption of the ovarian microenvironment, inhibit ovarian stromal vessels, follicular growth, an effective antioxidant enzymatic defense system, and the development of perifollicular vascularization (Tatone et al., 2014). The chain reactions that follow one another affect numerous physiological reactions in women and lead to reproductive disorders such as endometriosis, polycystic ovary syndrome, and unexplained infertility (Walsh et al. 2014).

Current studies have shown that antioxidants are also reliable and effective in improving infertility outcomes. Vitamin E has been studied for many years owing to its strong antioxidant effect, and use is being made of its therapeutic activity (Tongtako et al. 2018). Vitamin E is thought to act as a direct free radical scavenger by activating intracellular antioxidant enzymes and protecting cell membranes from lipid peroxidation occurring on cellular membrane components of the ovary (Traber and Atkinson 2007).

The limited numbers of studies of infertility caused by hyperthyroidism and treatment using antioxidant supplements show that comprehensive investigation is now required. Although there are studies indicating that hyperthyroidism affects fertility, these are both insufficient in number and inconsistent. We set out to prepare the ground for new molecular studies and to provide practical solutions to infertility problems caused by hyperthyroidism. We therefore investigated the effects of hyperthyroidism on the pituitary–ovarian axis by means of stereological, electron microscopic, and biochemical analyses, and by revealing the protective effect of vitamin E through its powerful antioxidant properties.

## Materials and methods

In total, 40 healthy female Wistar albino rats weighing 250–350 g and aged 10 weeks were used in this study. Approval was granted by the Ondokuz Mayıs University Animal Experiments Local Ethics Committee (no. 2017/52 dated 24 November 2017). The rats were obtained from the Ondokuz Mayıs University Experimental Animal Research Center and were housed in that center with a maximum of eight animals per cage during the experimental period. The living conditions during the study period were 22 ± 2 °C room temperature, 50 ± 10% relative humidity, and 12 h/12 h day and night photoperiods. The rats were allowed ad libitum access to standard rat chow (Bil-Yem, Ankara, Türkiye), and their water (~50 ml/day/rat) was changed daily.

### Formation of experimental groups

Groups of eight animals each were established. The number of animals to be used in the groups was determined by means of a power analysis test.

#### Group: Control group (Cont)

The animals in this group were not subjected to any treatment (*n* = 8).

#### Group: Sham

The animals in this group received 2 ml of corn oil by oral gavage at the same time (09:30–10:30) every day for 21 d (*n* = 8). This group was created to measure the stress factor of gavage application.

#### Group: Hyperthyroidism (Hypert) group

Animals in this group received L-thyroxine (Sigma Chemical Co., Dorset, UK) 0.3 mg/kg/day intraperitoneally at the same time (09:30–10:30) every day for 21 d (*n* = 8) (Atici et al. 2017).

#### Group: Hyperthyroidism + vitamin E group (Hypert + Vit E)

Animals in this group were given L-thyroxine (Sigma Chemical Co., Dorset, UK) 0.3 mg/kg/day intraperitoneally at the same time (09:30–10:30) every day for 21 d, and 100 mg/kg/day vitamin E (Sigma-258024) dissolved in corn oil orally by gavage (*n* = 8) (Sajitha et al. 2016).

#### Group: Vitamin E group (Vit E)

Animals in this group were given 100 mg/kg/day vitamin E dissolved in corn oil orally by gavage at the same time (09:30–10:30) every day for 21 d (*n* = 8). During the experimental period, animals were given standard rat chow ad libitum.

After the experimental procedures, the subjects were perfused transcardially under ketamine and xylazine 90/10 mg/kg anesthesia, and their ovarian tissues were removed.

#### Creation of the hyperthyroid model

In order to create the hyperthyroid model, L-thyroxine (Sigma Chemical Co., Dorset, UK) was first dissolved in 0.01 N NaOH and then in 0.9% NaCl to obtain a solution at a dose of 0.3 mg/kg. The prepared stock solution was stored at +4 °C (Baltaci and Mogulkoc, 2017).

### Histological methods

#### Light microscopic procedures

The rat ovarian tissues were placed into bottles containing 4% paraformaldehyde and 2% glutaraldehyde. The tracking procedures were performed after 15 d post-fixation. In order to perform the analyses in a blinded manner, each ovarian tissue was given a code number and embedded into paraffin blocks. Thick sections (25 μm) were taken from the paraffin blocks using a rotary microtome (Leica RM2125RT, Leica RM2125RT, Leica Biosystems, Nussloch GmbH, Nussloch, Germany) with a sampling interval of ½. These sections were stained with hematoxylin–eosin for stereological analyses.

#### Electron microscopic procedures

After 10 d of post-fixation in solutions containing 4% paraformaldehyde and 2% glutaraldehyde, ovarian tissues were processed and embedded in resin blocks (pure araldite, Araldite 502) for electron microscopic analysis. Semi-thin sections (0.5 µm) were cut using a rotary microtome (Leica RM 2135, Leica Instruments, Nussloch, Germany) and stained with toluidine blue. These semi-thin sections were then examined using a light microscope (Olympus BX43, Center Valley, PA, USA), and images were captured using a digital camera (Olympus SC50, Center Valley, PA, USA) connected to the microscope at magnifications of 20×, 40×, and 100× (NA 0.40; 0.65; 1.25 oil). In addition, 70 nm sections were cut, placed on 200 mesh grids, and stained with uranyl acetate/lead citrate for contrast enhancement. Electron microscopic images were taken using a transmission electron microscope (TEM; accelerating voltage for TEM: 80 kV; Jeol JSM-7001F, Japan). Once the appropriate tissue tracking procedures for scanning electron microscopic (SEM) analyses had been performed the ovarian tissues were coated with gold–palladium, an electron reflecting and electron-deflective material, to visualize their surfaces. SEM images were taken of the tissues (accelerating voltage for SEM: 5 kV; Jeol JSM-7001F, Japan).

### Stereological analyses

#### Estimation of follicle numbers using the optical fractionation method

**Fig. 1:**
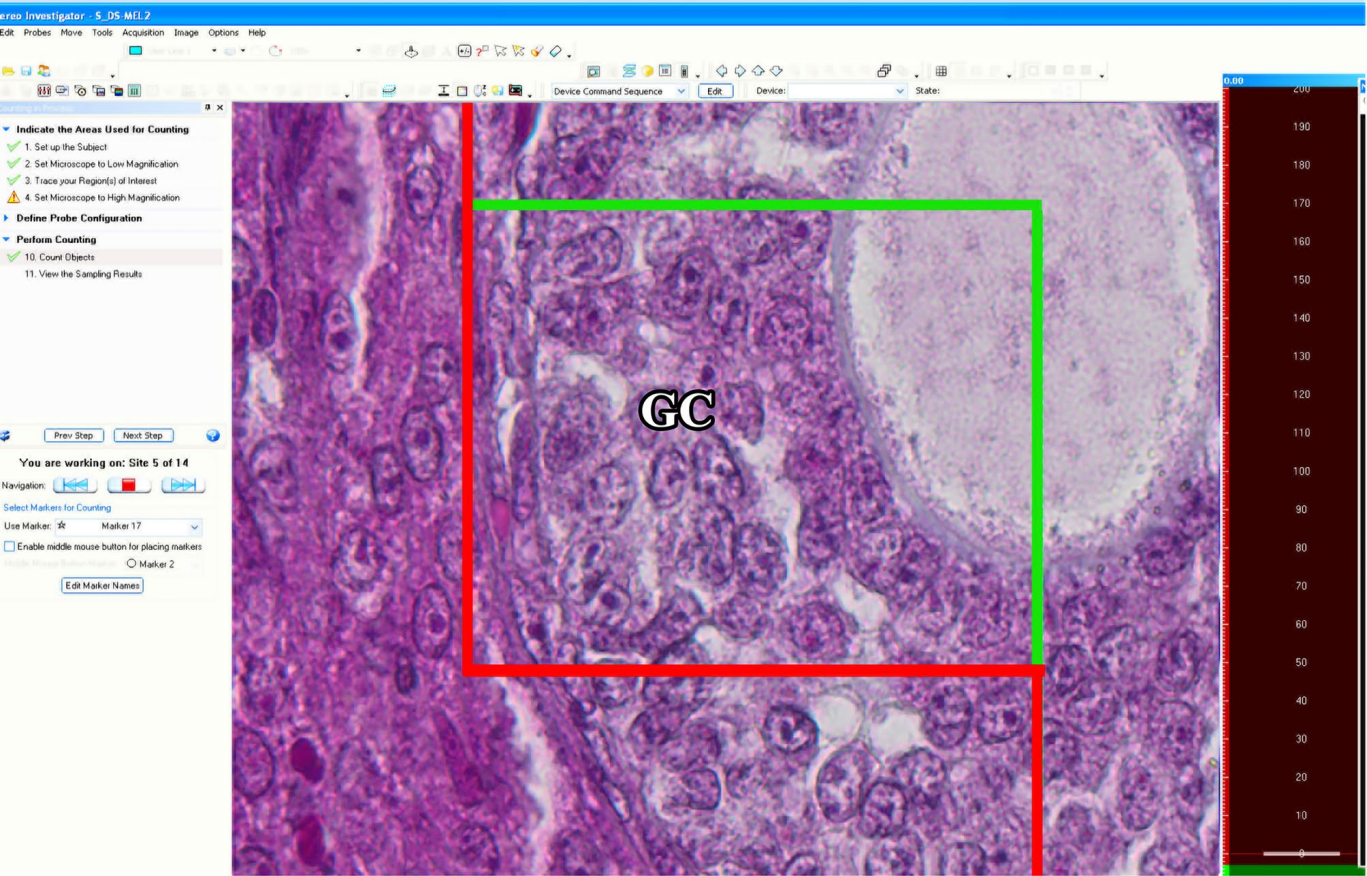
The optical fractionation method used for the counting of ovarian follicle numbers. If the nucleus of an oocyte is inside the unbiased counting frame, it is counted as a follicle. If the nucleus of an oocyte is outside or superimposed  with the red line of the unbiased counting frame, it is excluded from the follicle count. GC, granulosa cells

**Fig. 2:**
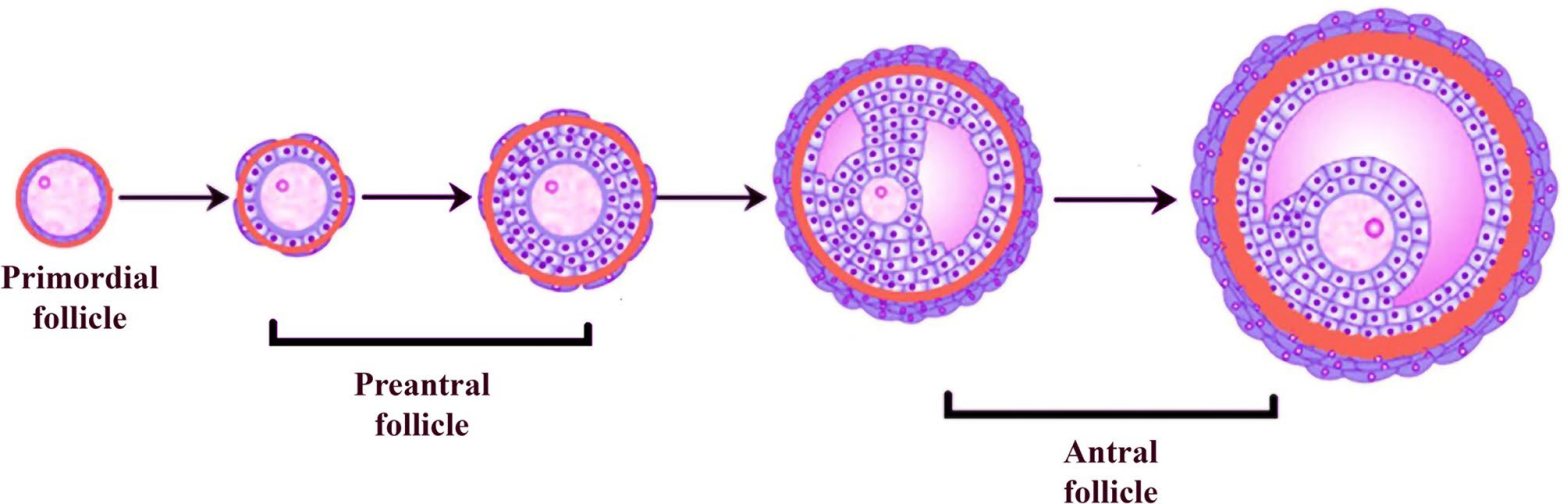
Classification of ovarian follicles (modified from Camaioni et al. 2018)

One part of the stereological analyses was performed on the images taken using a microscope equipped with Stereoinvestigator (MBF Biosciences, Williston, VT, USA) software at the Recep Tayyip Erdoğan University Department of Histology and Embryology (Fig. 1). The other part of the analysis was performed on the images taken with a light microscope with a digital color camera attachment (Leica DM 4000, Wetzlar; Germany) at the Ondokuz Mayıs University Department of Histology and Embryology. During the analysis, the ovarian follicles were classified on the basis of the classical follicle classification (Pedersen and Peters, 1968). In this classification, the shape of granulosa cells and the number of cell layers were considered on the basis of a previous study (Camaioni et al. 2018) (Fig. 2).

Ovarian follicles were counted in the stereology analysis system using the optical fractionation method. Transverse sections were taken from each ovarian tissue at a thickness of 25 μm and a sampling interval of ½, in accordance with the systematic random sampling (SRS) rule. After the sectioning process, an average of 35–40 sections were obtained from each ovarian tissue. The parameters used in this research were determined by a pilot study. In accordance with that pilot study, the area of the unbiased counting frame was determined as 6400 μm^2^, the step interval as 40,000 μm^2^, and the area sampling ratio as 6400 μm^2^/40,000 μm^2^. The guard zone was 3 μm for the upper part of the section, and the disector height was 10 μm.

#### Volume estimation

Cortex, medulla, preantral follicle, antral follicle, corpus luteum, connective tissue and vascular volumes, and the cortex/medulla volume ratio were calculated using the Cavalieri method on 25 μm sections taken with ½ sampling from each ovarian tissue from all groups. The point density on the point counting grid was designed to obtain an appropriate error coefficient (CE). Coefficient of variation (CV) and CE values were calculated (Gundersen and Jensen 1987; Altun et al. 2024). The total number of test points in the grid corresponding to the relevant areas was counted. The following formulae were then used to estimate the volume.

$$V_{ref} = \sum Pi \times \left( {a/p} \right) \times t$$

where “*V*_ref_” is the total or reference volume of the relevant structure; “∑Pi” the total number of superimposed points on the cross-sectional surface area; “*a*/*p*” the point area representing the area represented by a point on the grid; and “*t*” the cross-sectional thickness (Kaya et al. 2024). After applying the same formula to all sections, The total volume value of a single ovarian tissue was calculated after applying the same formula to all sections.

$$\sum {\mathrm{V}}_{{{\mathrm{ref}}}} = {\mathrm{V1}} + {\mathrm{V2}} + {\mathrm{V3}} + \cdots \cdots \cdots \cdots \cdots$$

### Biochemical analyses

Blood samples before sacrification were collected from the right atrium of all animals with the help of a syringe and placed into tubes. They were centrifuged at 2000 × *g* for 15 min and stored in a refrigerator at −20 °C. All biochemical analyses were performed at the university biochemistry laboratory.

#### TSH and FT4 hormone analysis

TSH (catalog no. 201–11-0181) and ST4 Sunredbio (catalog no. 201-11-0736) measurement kits were used to determine the animals’ hyperthyroidism status. For this purpose, the solutions in the kit were prepared before starting the analysis. The plates were filled with serum and other solutions according to the kit protocol. Once the enzyme in the plates had reacted with the serum, spectrophotometric measurements were taken at a 450 nm wavelength.

#### FSH and LH hormone analysis

FSH (catalog no. 201-11-0449) and LH (catalog no. 201-11-0180) Sunredbio kits were used to show the effects of hyperthyroidism on hormonal levels. The solutions in the kit were prepared before starting the analysis. The plates were first filled with serum and other solutions in accordance with the kit protocol. Once the enzyme in the plates had reacted with the serum, spectrophotometric measurements were taken at a 450 nm wavelength.

#### Catalase

A catalase (CAT) (Cayman, 707,002) measurement kit was employed to show CAT enzyme activity. The solutions in the kit were prepared before starting the analysis. The plates were first filled with serum and other solutions in accordance with the kit protocol. After the enzyme in the plates had reacted with the serum, spectrophotometric measurements were performed at 450 nm.

#### Superoxide dismutase

We used a superoxide dismutase (SOD) (Bioscience, 706,002) measurement kit to determine SOD enzyme levels. The solutions in the kit were first prepared before starting the analysis. The plates were filled with serum and other solutions in line with the instructions. After the enzyme in the plates reacted with the serum, spectrophotometric measurements were performed at 450 nm.

## Statistical analysis

The study data were analyzed on SPSS software (SPSS version 21.0; SPSS Inc., Chicago, IL, USA). The data were expressed as mean ± standard deviation. Normality and homogeneity tests showed that all groups exhibited normal distribution. Differences between the groups were evaluated using one-way analysis of variance (ANOVA) and Tukey’s test. In addition, once the normality and homogeneity tests for the pre-experiment and post-experiment measurements had been performed, the differences between the groups were evaluated using the paired sample *t*-test. *p*-Values < 0.05 were considered statistically significant.

## Results

### Stereological findings

#### Findings obtained by the optical fractionation method

**Fig. 3:**
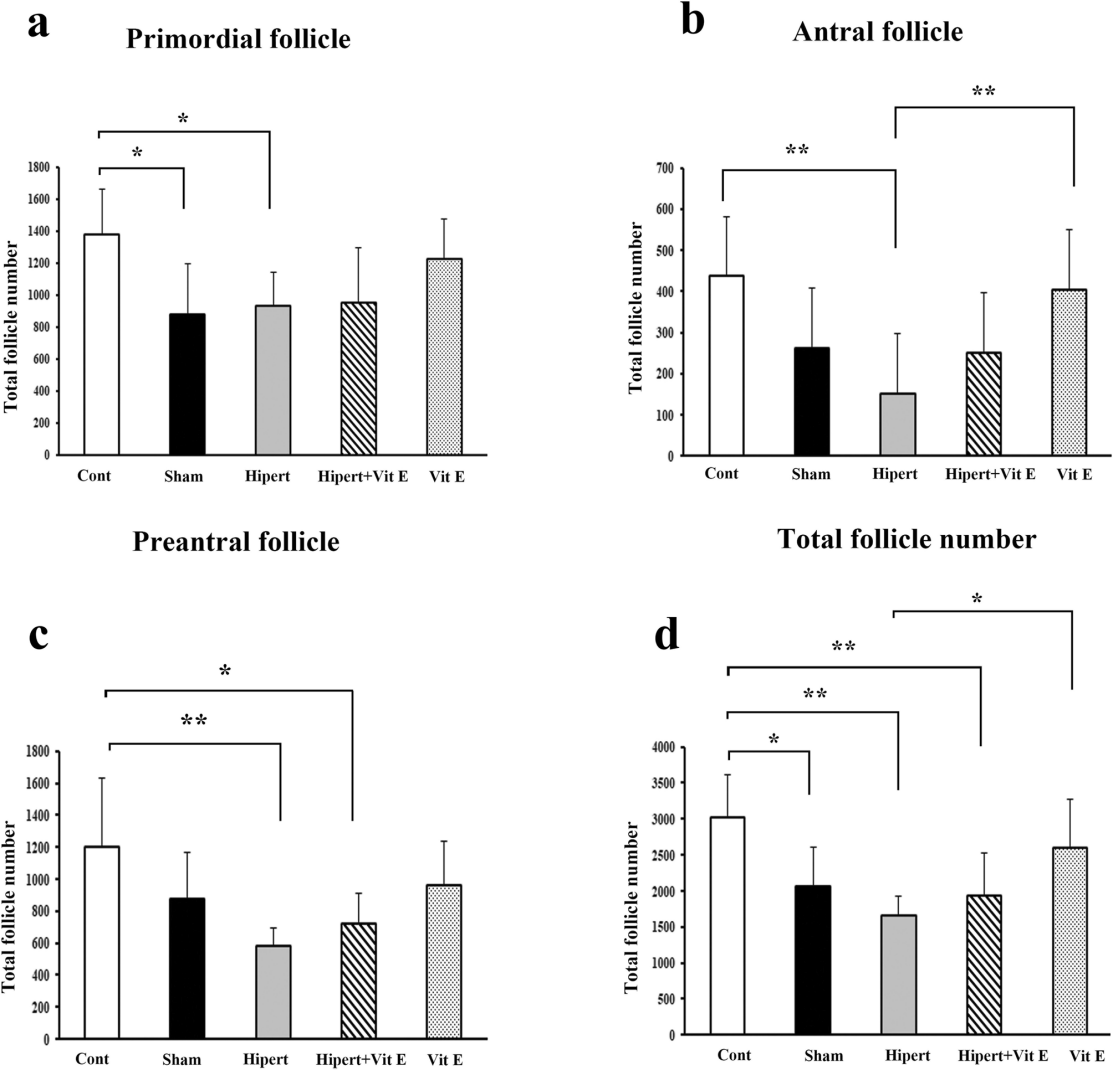
Total **a** primordial, **b** preantral, **c** antral, and **d** total follicle numbers of each group. Primordial, preantral, antral, and total follicle counts were statistically significantly decreased in the Hypert group compared with the Cont group. Statistically significant differences at the *p* < 0.05 and *p* < 0.01 levels are indicated by (*) and (**), respectively

The total primordial, preantral, antral, and total follicle numbers in the ovarian tissues from all the study groups are shown in Fig. 3.

As a result of the statistical evaluation of the data obtained from the stereological analysis of the total primordial follicle numbers in all groups: it was observed that the number of primordial follicles significantly decreased in the Hypert and Sham groups compared with the Cont group (*p* < 0.05). When the Hypert + Vit E and Hypert groups were evaluated in terms of primordial follicle number, it was observed that although an increase was observed in the Hypert+ Vit E group, this increase was not statistically significant (*p* > 0.05).

In the stereological analysis results of the total preantral follicle numbers: it was observed that the number of preantral follicles significantly decreased in the Hypert group compared with the Cont group (*p* < 0.01). Similarly, it was determined that the number of preantral follicles in the Hypert+ Vit E group significantly decreased compared with the Cont group (*p* < 0.05). When the Hypert and Hypert + Vit E groups were compared, it was observed that the number of preantral follicles in the Hypert+ Vit E group increased compared with the Hypert group; however, this increase was not statistically significant (*p* > 0.05).

In the stereological analysis of antral follicle numbers, it was observed that the number of antral follicles decreased significantly in the Hypert group compared with the Cont and Vit E groups (*p* < 0.01). In addition, it was observed that the number of antral follicles in the Sham and Hypert groups decreased compared with the Cont group; however, these decreases were not statistically significant (*p* > 0.05). When the Hypert+ Vit E group was compared with the other groups, no significant change was detected in the number of antral follicles (*p* > 0.05).

As a result of the statistical evaluation of the data obtained from the stereological analysis of the total follicle numbers in all groups, a statistically significant decrease was detected in the Sham group compared with the Cont group (*p* < 0.05). It was observed that the total follicle number decreased statistically significantly in both the Hypert group and the Hypert+ Vit E group compared with the Cont group (*p* < 0.01). When the Vit E group was compared with the other groups (Cont, Sham, and Hypert + Vit E), no statistically significant change was found in total follicle numbers (*p* > 0.05).

#### Volume estimation

**Fig. 4:**
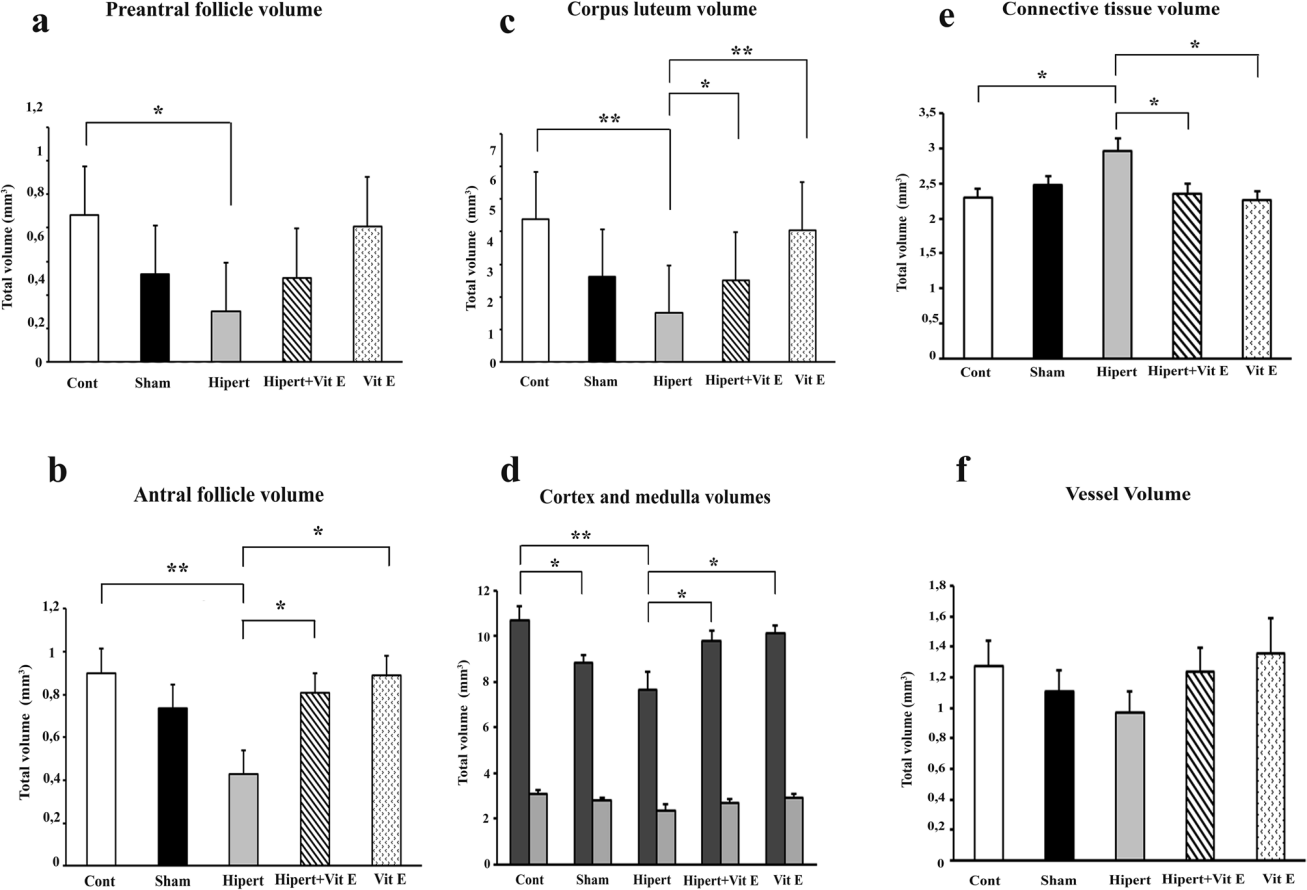
Differences in **a** preantral and **b** antral follicle, **c** corpus luteum, **d** cortex and medulla, **e** connective tissue, and **f** vascular volumes between the groups following the creation of the experimental model are shown in the graphs. Preantral follicle, antral follicle, corpus luteum, and cortex volumes were statistically significantly decreased in the Hypert group compared with the Cont group. Connective tissue volume was significantly increased in Hypert group compared with the Cont, Hypert + Vit E, and Vit E groups. Differences that are statistically significant at *p* < 0.05 are indicated by (*), and those significant at *p* < 0.01 are indicated by (**)

The preantral and antral follicle, corpus luteum, cortex, medulla, connective tissue, and vascular tissue volumes in the ovarian tissues from all the study groups are shown in Fig. 4.

As a result of the statistical evaluation of the volumetric analyses of preantral follicles calculated with the Cavalieri method in all groups of our study, a statistically significant decrease was observed in the Hypert group compared with the Cont group (*p* < 0.05). When the Cont group was compared with the other groups (Sham, Hypert, and Hypert + Vit E), no statistically significant difference was found (*p* > 0.05).

In the results of the volumetric analyses of antral follicles, a statistically significant decrease was observed in the Hypert group compared with the Cont group (*p* < 0.01). Similarly, it was determined that the antral follicle volume showed a statistically significant decrease in the Hypert group compared with the Vit E group (*p* < 0.05). When the Hypert + Vit E group was compared with the Hypert group, it was determined that the antral follicle volumes increased statistically (*p* < 0.05). When the Cont and Sham groups were compared with the Vit E group, no statistically significant difference was found (*p* > 0.05).

In the volumetric analyses of corpus luteums, a statistically significant decrease was observed in the Hypert group compared with the Cont and Vit E groups at the level of *p* < 0.01; while in the Hypert + Vit E group, a statistically significant increase was observed in the Hypert group at the level of *p* < 0.05. When the Cont and Sham groups were compared with the Vit E group, no statistically significant differences were found in corpus luteum volumes (*p* > 0.05).

As a result of the statistical evaluation of the cortex volumetric analyses calculated with the Cavalieri method of the ovarian tissues in all groups of our study, a statistically significant decrease in cortex volume was observed in the Hypert group compared with the Cont group (*p* < 0.01), while a statistically significant increase was observed in the Hypert + Vit E group compared with the Hypert group (*p* < 0.05). When all groups were evaluated in terms of medulla volume values, no statistically significant differences were found between the groups (*p* > 0.05).

As a result of the statistical evaluation of the connective tissue volumetric analyses calculated with the Cavalieri method, a statistically significant increase was observed in the Hypert group compared with the Cont, Hypert + Vit E, and Vit E groups (*p* < 0.05). When the Cont and Sham groups were compared with the Vit E group, no statistically significant difference was found (*p* > 0.05).

In the vascular volume analyses calculated with the Cavalieri method, a decrease was observed in the Hypert group compared with the Cont, Sham, Hypert + Vit E, and Vit E groups, but this decrease was not found to be statistically significant (*p* > 0.05).

### Biochemical results

**Fig. 5:**
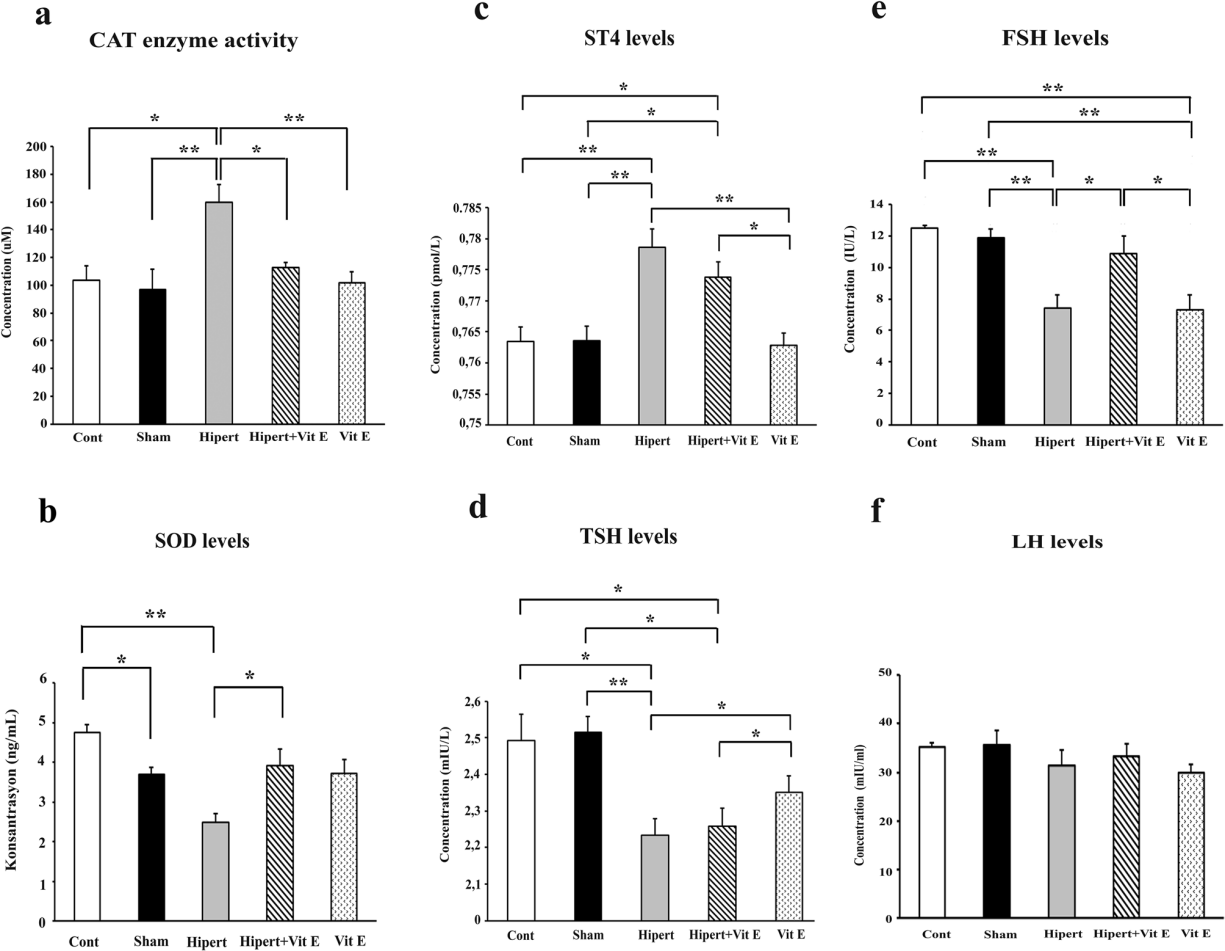
Graphs showing **a** CAT enzyme activity, **b** SOD enzyme levels, and **c** ST4, **d** TSH, **e** FSH, and **f** LH hormone levels in all the groups. In the Hypert group, CAT enzyme activity increased significantly compared with the Cont, Sham, and Hyper + Vit E groups, while SOD levels decreased. In the Hypert group, ST4 values increased significantly compared with the Cont, Sham, Hyper + Vit E, and Vit E groups, while TSH levels decreased. Differences significant at *p* < 0.05 are indicated by (*), and those significant at *p* < 0.01 by (**)

SOD enzyme levels, CAT enzyme activity, and TSH, ST4, FSH, and LH hormone values were measured in blood serum samples collected from members of all the groups. The findings are presented in Fig. 5.

In the evaluation of CAT enzyme activity in each of the groups, it was determined that CAT enzyme activity increased significantly in the Hypert group compared with the Cont and Hypert + Vit E groups (*p* < 0.05). At the same time, it was observed that CAT enzyme activity in the Hypert group showed a statistically significant increase compared with the Sham and Vit E groups (*p* ≤ 0.01).

In the comparison of SOD enzyme levels between the groups, it was determined that SOD enzyme levels decreased significantly in the Cont and Hypert + Vit E groups compared with the Hypert group (*p* < 0.01). Similarly, no significant difference was observed between the Cont and Sham groups when compared with the Vit E group (*p* > 0.05).

When the TSH hormone values in the blood serum samples were compared, it was determined that TSH values in the Hypert group showed a statistically significant decrease compared with the Sham group (*p* < 0.01). Similarly, it was observed that the TSH values of the Hypert group decreased statistically significantly compared with the Cont and Vit E groups (*p* < 0.05). It was determined that the TSH values ​​in the Hypert + Vit E group decreased statistically significantly compared with the Cont, Sham, and Vit E groups (*p* < 0.05). Conversely, when the Hypert group was compared with the Hypert + Vit E group, no statistically significant difference was found (*p* > 0.05).

In the evaluation of the groups in terms of ST4 hormone values, it was determined that the ST4 hormone values in the Hypert group showed a statistically significant increase compared with the Cont, Sham, and Vit E groups (*p* < 0.01). It was determined that the ST4 values increased statistically significantly in the Hypert + Vit E group compared with the Cont, Sham, and Vit E groups (*p* < 0.05). However, when the Hypert group was compared with the Hypert + Vit E group, no statistically significant difference was found (*p* > 0.05).

When the FSH hormone amounts obtained from the blood serum samples were evaluated, it was observed that the FSH levels in the Hypert and Vit E groups decreased statistically significantly compared with the Cont and Sham groups (*p* < 0.01). When the Hypert + Vit E group was compared with the Cont and Sham groups, no statistically significant difference was observed (*p* > 0.05); however, when compared with the Hypert and Vit E groups, a statistically significant increase in FSH levels was observed (*p* < 0.05). In the comparison of the LH hormone levels obtained from blood serum samples between the groups, it was observed that the LH level in the Hypert group decreased compared with the Cont, Sham, and Hypert + Vit E groups; however, this decrease was not statistically significant (*p* > 0.05). Similarly, it was determined that the LH levels increased in the Hypert + Vit E group compared with the Hypert group; however, this increase was not statistically significant (*p* > 0.05).

### Histopathological findings

#### Light microscopic findings obtained from semi-thin sections

**Fig. 6:**
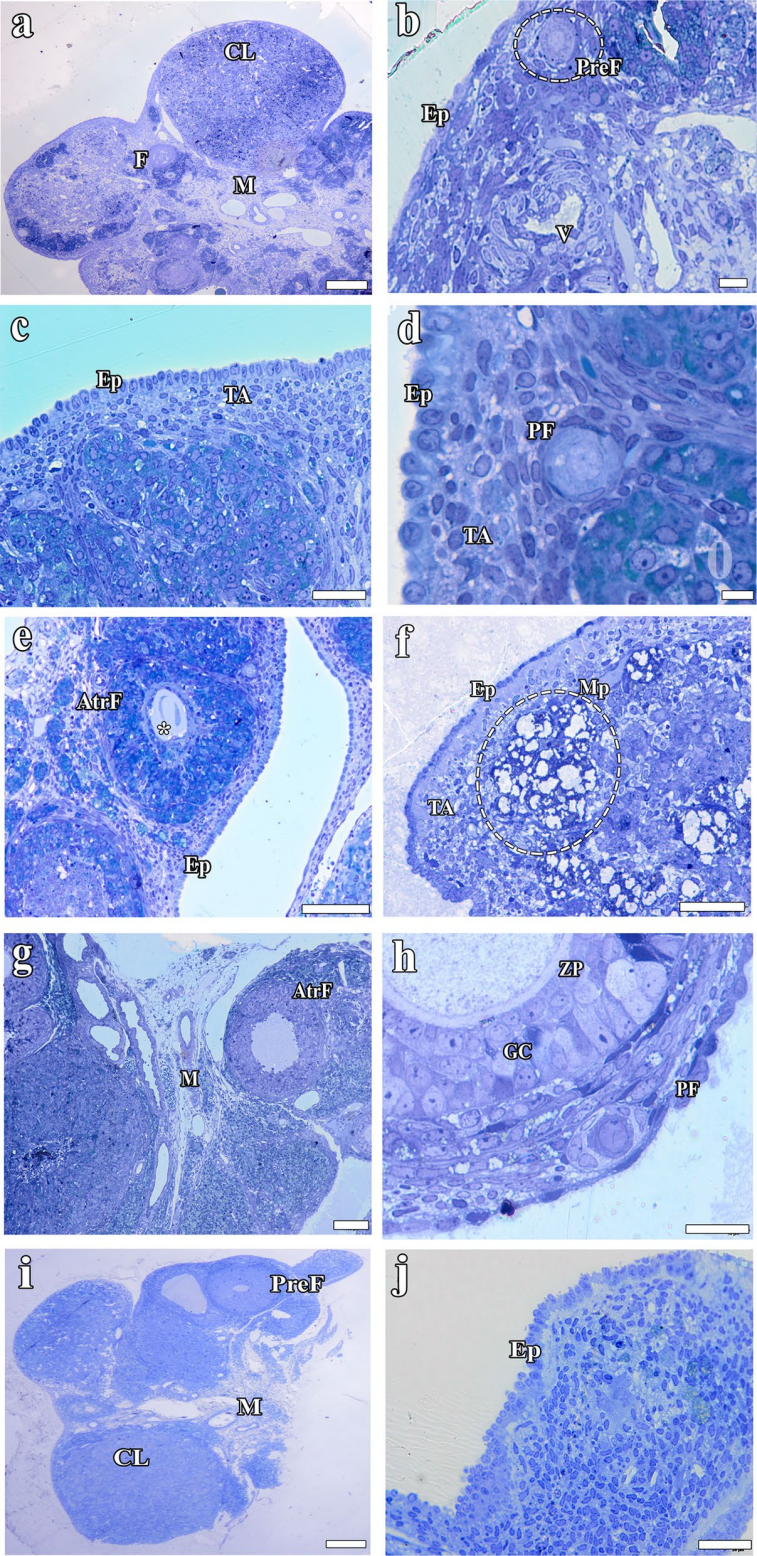
Light microscopic images of semi-thin tissue sections: **a**, **b** Cont; **c**, **d** Sham; **e**, **f** Hypert; **g**, **h** Hypert + Vit E; and **i**, **j** Vit E groups. AtrF, atretic follicle; (*), degenerated oocyte; V, vessel; CL, corpus luteum; Ep, covering epithelium; M, medulla; Mp, macrophage populations; PF, primordial follicle; PreF, preantral follicle; GC, granulosa cells; TF, theca follicle; ZP, zona pellucida. Scale bars **a**, **c**, **e**, **f**, and **g** = 100 μm; **i** = 200 μm; **b**, **d**, **h**, and**j** = 20 μm

**Fig. 7:**
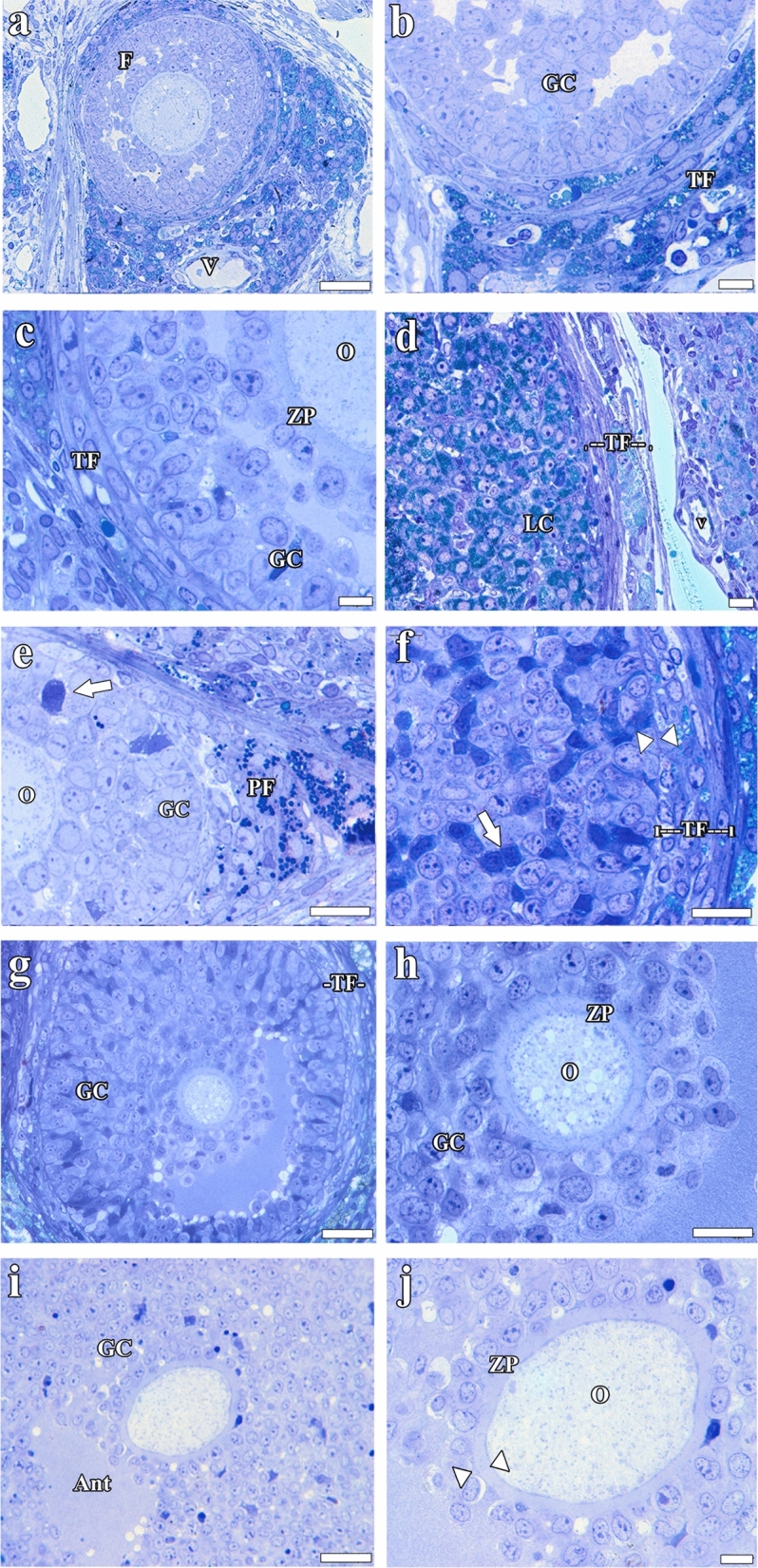
Light microscopic images of semi-thin tissue sections: **a**, **b** Cont; **c**, **d** Sham; **e**, **f** Hypert; **g**, **h** Hypert + Vit E; and **i**, **j** Vit E. Granulosa cells undergoing apoptosis (white arrow); basement membrane (arrowhead). Ant, antrum; F, follicle; GC, granulosa cells; LC, lutein cells; TF, theca follicle; O, oocyte; ZP, zona pellucida. Scale bars: **b**, **c**, and *j* = 10 μm; **f** and **h** = 20 μm; **a**, **d**, **e**, **g**, and **i** = 40 μm

The epithelial cells of ovarian tissues from the Cont, Sham, and Vit E groups exhibited a normal structure, and the tunica albuginea was clearly visible (Fig. 6 a–d, g–j). The follicles exhibited a normal tissue structure, and a widespread blood vessel network was observed in the surrounding tissues (Fig. 6 a–d, g–j). The borders of the oocyte in the follicle were clear and distinct, and the zona pellucida surrounding it exhibited a prominent structure in the Cont, Sham, and Vit E groups (Fig. 7 a–d, g–j). At the same time, the theca follicle was well developed around the corpus luteum formed after ovulation, and the lutein cells exhibited a normal morphology (Fig. 7 a–d, g–j). In the ovarian tissues from the Hypert group, the single-layered epithelium surrounding the outer surface was cuboidal in some areas and flattened in others, and numerous dark-stained cells were observed among the epithelial cells (Fig. 6 e, f). The tunica albuginea under the epithelium was thin and its borders were indistinct. Numerous large-scale macrophage clusters were observed in the areas following the tunica albuginea (Fig. 6f). Dark-stained granulosum cells, undergoing apoptosis, were observed in the wall of the preantral follicle (Fig. 7 e, f). The zona pellucida surrounding the oocyte in the follicle center was remarkably thin (Fig. 7e). The presence of numerous dark-stained cells may show that the degeneration was severe. The borders of the follicle cells were indistinct, the cell nuclei were relatively small, and many atretic follicles were seen in the ovarian cortex (Fig. 7f). While the theca follicle surrounding the follicle was visible, the capillaries in the theca interna and the glassy membrane separating the granulosum cell layer from the theca follicle were not well developed (Fig. 7f).

#### Electron microscopic findings

##### Transmission electron microscopic image findings

**Fig. 8:**
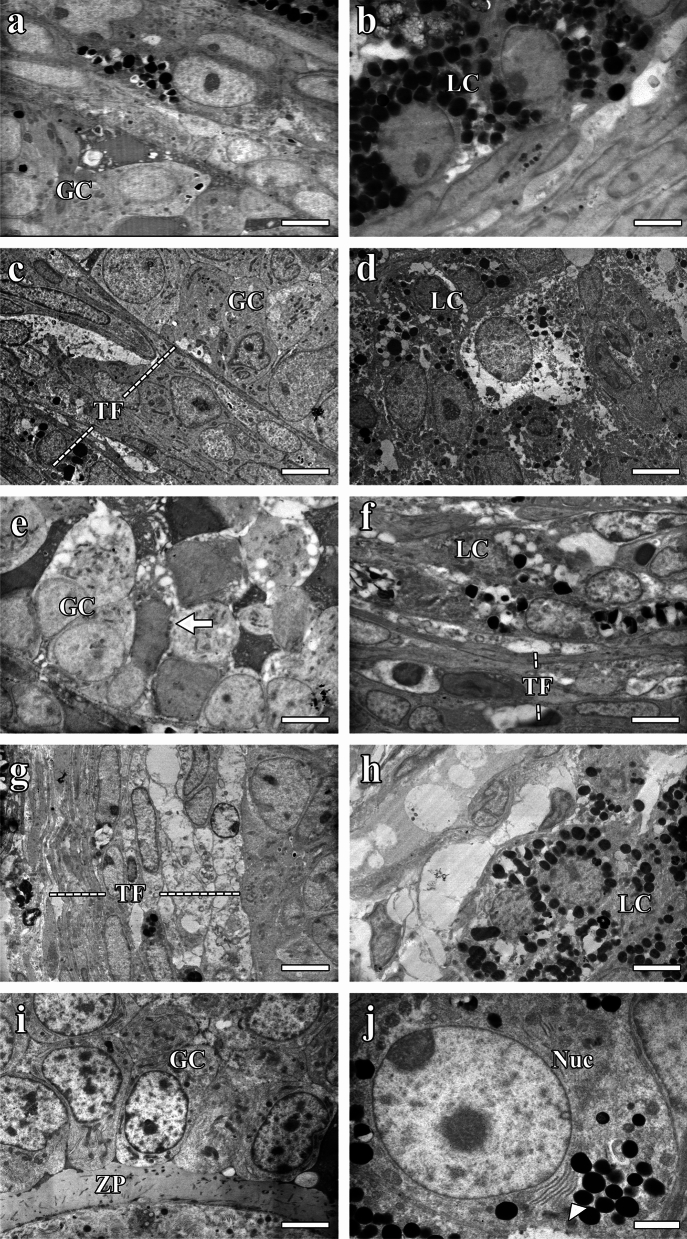
TEM images: **a**, **b** Cont; **c**, **d** Sham; **e**, **f** Hypert; **g**, **h**, Hypert + Vit E; and **i**, **j** Vit E. Granulosa cells undergoing apoptosis (white arrow) and Golgi complex (arrowhead). GC, granulosa cells; LC, lutein cells; Nuc, nucleus; TF, theca follicle; ZP, zona pellucida. Scale bars: **a**–**i** = 4 μm, **j** = 2 μm

Examination of the TEM images of ovarian tissues from the Cont, Sham, Hypert + Vit E, and Vit E groups revealed a normal cell content in the granulosum and lutein cells in the ovarian tissue, and the boundaries of the cell nuclei were easily distinguished (Fig. 8 a–d, g–j). The lipid droplets in the lutein cells from the Cont, Sham, and Vit E groups were of different sizes and the theca follicles exhibited a normal structure, their internal and external layers being clearly differentiated (Fig. 8 b, c, g). The cells in the corpus luteum structure in the Hypert + Vit E group contained numerous lipid droplets of different sizes, and the cell cytoplasm and blood vessels were partially preserved (Fig. 8 g, h). Both the granulosa cells and the theca follicle were well preserved, and the cell boundaries were distinct (Fig. 8 g, h). In the Vit E group, the lutein cells exhibited numerous lipid droplets with well-preserved boundaries, and the Golgi complex, nuclear membrane, nucleolus, and agranular endoplasmic reticulum were healthy in appearance (Fig. 8j). Electron microscopic examination of the ovarian tissues from the Hypert group revealed that the cell and nuclear borders were unclear in most of the granulosum cells. Dark-stained cells that had largely lost their functions were observed between these cells (Fig. 8e). The cytoplasm and nuclei of the granulosum were darker, and there were numerous gaps within and between the cells (Fig. 8e). Some cells in the theca follicle had lost their cytoplasm, and empty vesicular structures were common in the cytoplasm of cells containing lipid droplets (Fig. 8f). Numerous lipid droplets with distinct boundaries were observed in the cytoplasm of the luteal cells (Fig. 8f). The lumens of the capillaries in the theca interna were filled with an amorphous substance (Fig. 8f).

#### Scanning electron microscopic image findings

**Fig. 9:**
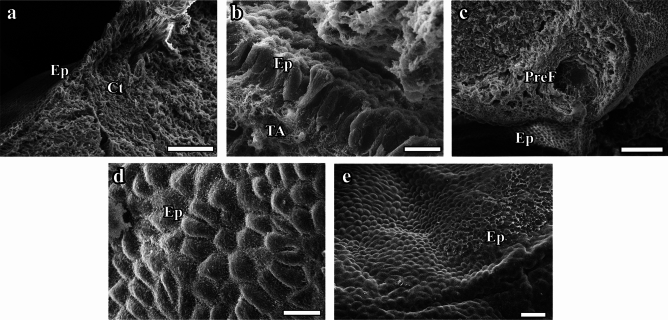
SEM images: **a**, **b** Cont; **c**, **d** Sham; **e**, **f**, Hypert; **g**, **h** Hypert + Vit E; and **i**, **j**, Vit E. Ep, covering epithelium; PreF, preantral follicle. Scale bars: **b** and **d** = 10 μm, **a** = 20 μm, **e** = 30 μm, **c** = 50 μm

Examination of SEM images of ovarian tissues from the Cont, Sham, and Vit E groups showed that the surface epithelium of the ovary was preserved and the intercellular space was normal (Fig. 9 a, b, d, e). The connective tissue and other structures under the epithelium were also normal in appearance (Fig. 9 a, b). The corpus luteum exhibited regular borders, with prominent lutein cells (Fig. 9 a, b). The outer surface of the organ maintained its integrity (Fig. 9 a, b, d, e). SEM images of the ovarian tissues from the Hypert + Vit E group showed a well-preserved surface epithelium of the ovary, a normal intercellular space, and the presence of cytoplasmic extensions of different widths and lengths at the apex (Fig. 9d). All follicles and vessels observed in the ovary had preserved their morphological structures. The SEM images from the Hypert group revealed that the borders of the epithelial cells covering the ovary were indistinct, and the cell height had decreased (Fig. 9c). The preantral follicle located immediately below the epithelium in the ovary had lost its integrity with the surrounding tissue, and the gaps between the follicle and the surrounding tissue had widened significantly (Fig. 9c).

## Discussion

This study set out to investigate the adverse effects of hyperthyroidism on the pituitary–ovarian axis in female infertility using stereological, biochemical, and histopathological approaches, and whether vitamin E, with its powerful antioxidant property, exerts a protective effect on the ovary. The study findings showed that hyperthyroidism produces destructive effects in ovarian tissue by reducing the number and volume of follicles and disrupting the structure of tissue components, eventually leading to infertility problems. However, vitamin E exhibited protective effects in ovarian tissue against hormonal imbalance and oxidative stress caused by hyperthyroidism.

In order to verify the hyperthyroidism, animal model created by L-thyroxine administration, TSH and ST4 hormone levels were evaluated in the serum samples. That analysis determined a statistically significant decrease in TSH values in the Hypert and Hypert + Vit E groups compared with the Cont, Sham, and Vit E groups. ST4 levels were higher in the Hypert and Hypert + Vit E groups compared with the Cont, Sham, and Vit E groups. The data obtained confirmed that the hyperthyroid model was successfully established.

Thyroid hormones play a critical role in the development and maturation of ovarian follicles and in the protection of various endocrine functions. Follicle numbers can therefore be evaluated as an important indicator of the effects of thyroid hormones on the ovary. Stereological evaluation in the present study revealed a significant decrease in the numbers of primordial, preantral, antral, and total follicles in the Hypert group compared with the Cont group. Preantral follicle, antral follicle, and corpus luteum volumes were significantly lower in the Hypert group compared with the Cont group. In a study conducted to demonstrate the relationship between thyroid dysfunction and ovarian reserve, the authors observed a significant decrease in ovarian size and capsule thickness, as well as primary and secondary follicle sizes, in a hyperthyroid group (Mahmud et al. 2021).

The findings of this study were consistent with those of previous research showing that hyperthyroidism reduces the total number of follicles in the ovary (Kabodmehri et al. 2021; Kaplan et al. 2021). Very few studies have investigated the effects of hyperthyroidism on ovarian follicle numbers. Further, more advanced studies presenting quantitative data concerning the effects of hyperthyroidism on ovarian follicle numbers are now needed.

The mechanisms underlying the adverse effects of hyperthyroidism on the ovary have not yet been comprehensively elucidated. Xu et al. (2020) showed that NOS1, NOS2, and NOS3 were expressed in surface oocytes, granulosa cells, and theca cells as a result of immunohistochemical ovarian tissue staining. Those authors also suggested that dysregulation of thyroid hormones affects NOS expression, and that these hormones may therefore regulate basal functions in ovarian follicular development. Zheng et al. (2015) reported that hyperthyroidism caused a decrease in the number of large antral follicles compared with a control group by altering ovarian NOS activity in a hyperthyroid model. Despite using different pathways, studies confirm that hyperthyroidism affects follicle development, either directly or indirectly. Future researches should focus on the underlying mechanisms, which should be confirmed using more comprehensive methods.

Volumetric analyses of the ovarian cortex using the Cavalieri method revealed an increased cortex volume in the Hypert group compared with the Cont group. No significant differences were determined between the groups in terms of vascular volumes. We encountered no previous study in which hyperthyroidism was evaluated in terms of ovarian connective tissue and vascular volume using stereological methods. The absence of any change in vascular volume in this study may perhaps be attributable to the dose of L-thyroxine applied to create the hyperthyroid model being insufficient to cause an effect on vascular tissue volume. Further studies involving different doses of L-thyroxine are now needed to clarify this issue.

Studies have shown that hyperthyroidism and hypothyroidism cause hormonal levels to change by disrupting the balance in the hypothalamic–ovarian axis, and this significantly affects follicular development (Colella et al. 2020). We observed a decrease in FSH levels in the Hypert group compared with the Cont group, but no significant change in LH levels. Hyperthyroidism reduces FSH secretion, adversely affects follicle development in the ovary, and causes a decrease in follicle numbers and volumes. The inhibitory effects on follicles in the current study suggest that the decrease in follicle numbers and FSH levels may have occurred because of hypothalamic–pituitary–gonadal (HPG) axis disruption due to hyperthyroidism.

A study examining the effect of experimentally-induced hyperthyroidism on reproductive dysfunction and gonadotropin secretion in female rats reported decreased serum TSH, FSH, and PRL concentrations, as well as pituitary FSHβ and TSH gene expression levels, in hyperthyroid animals. The authors concluded that altered gonadotropin secretion was associated with reproductive failure (Al-Saaidi and Al-Hasan 2022). However, the mechanisms of the increase or decrease in serum FSH and LH levels in hyperthyroidism have not been fully elucidated. Liu et al. (2018) investigated the effects of thyroid hormone irregularity on reproductive hormones in rats. Those authors reported significantly decreased serum gonadotropin-releasing hormone (GnRH) and FSH levels in both hyperthyroid and hypothyroid rat models compared with a control group, but no change in LH hormone levels (Liu et al. 2018). However, a study investigating the role of thyroid hormones on the female reproductive system observed significantly increased FSH hormone levels and significantly decreased LH levels in a rat hyperthyroid model compared with a control group (Wei et al. 2018). While our results are consistent with the results of many previous studies, they also differ in some respects. On the basis of these contradictory results in the literature, we therefore think that the effect of hyperthyroidism on pituitary hormones should be investigated comprehensively by widening the biochemical parameters examined.

Antioxidants such as SOD, CAT, and glutathione peroxidase (GPx) are directly related to oocyte quality and are important biomarkers in assisted reproduction techniques. Thyroid hormones play an important role in the regulation of basal metabolic status. They therefore exhibit important effects on antioxidant capacity (such as SOD, CAT, and GPx) and oxidative metabolism (Bozbek and Şentürk 2023). In hyperthyroidism induced by thyroxine or triiodothyronine administration, the rise in metabolic rate increases microsomal oxidative capacity and free radical formation (Nosratzehi et al. 2024). Similarly, the decrease in SOD levels and increase in CAT activity observed in hyperthyroid animals may show that hyperthyroidism causes oxidative stress by disrupting the antioxidant balance in tissues.

Costilla et al. (2019) reported an increase in CAT and GPx-1 expression together with increased ROS due to hyperthyroidism. Increased ROS was shown to induce antioxidant enzyme transcription through the activation of Nrf-2 factor in tissues due to hyperthyroidism (Costilla et al. 2019). Nrf-2 is localized in the cytoplasm under normal conditions, but under oxidative stress conditions, it migrates to the nucleus to increase the expression of genes related to antioxidant response elements in the Nrf-2 promoter region. The activated Nrf-2 factor stimulates the transcription of enzymatic antioxidants. It can therefore be suggested that the increase in CAT activity observed in the Hypert group may derive from the mechanism mentioned above. A decrease in SOD levels and increase in CAT activity due to hyperthyroidism observed in this study may be owing to hyperthyroidism causing superoxide accumulation, which induces oxidative stress and prevents follicle development.

Under pathophysiological conditions, excessive ROS causes a decrease in antioxidant levels in oocytes, cumulus cells, and follicular fluid, and consequently a decrease in ovarian follicle numbers and oocyte quality (Cosme et al. 2023). Wei et al. (2018) observed that hyperthyroidism changes the antioxidant status by increasing the amount of NO and NOS activity in the ovary. At the same time, they determined that secondary and antral follicle and corpus luteum numbers in the ovaries of animals with this condition, which develops due to oxidative stress, were significantly reduced compared with the euthyroid group (Wei et al. 2018). A rise in NO levels causes an increase in cytokine levels and activation of nuclear factor-kappa B, which induces ROS production and is also an important biomarker in ovarian tissue damage (Kaygusuzoglu et al. 2018). Changes in the majority of the biochemical parameters examined associated with oxidative stress and female ovarian antioxidant defense enzymes support our biological comments. It may be suggested that the changes observed in SOD and CAT activity are due to an adaptive response against oxidative stress aimed at neutralizing increased superoxide radicals and hydrogen peroxide because of the hypermetabolic state caused by hyperthyroidism. To summarize, the SOD and CAT activity values in this study show that hyperthyroidism induces oxidative stress, which disrupts the hormonal balance of the body and causes ovarian damage.

The findings of decreased cortex volume and increased connective tissue volume in the ovarian tissues in this study are similar to the pathological features of ovarian fibrosis (a primary thick capsule, increased connective tissue, and decreased or absent follicles) (Lan et al. 2024). Patients with ovarian fibrosis are susceptible to infertility and tend to reduce their response to fertility treatment. Transforming growth factor-β1 (TGF-β1) is a cytokine that plays an important role in the formation and development of fibrosis. Studies have shown that hyperthyroidism increases tissue TGF-β1 levels (Sviridenko et al. 2022). Although no research to date has associated ovarian fibrosis with hyperthyroidism, further studies are needed to fully elucidate this.

Vitamin E scavenges free oxygen radicals and thus provides protection against oxidative stress. Therefore, under various pathological conditions, vitamin E reduces the damage to histological structures in ovarian tissue. The biochemical results of this study showed that SOD enzyme levels and CAT enzyme activity in the Hypert + Vit E group were within normal ranges. Sargazi et al. (2019) evaluated the protective effect of vitamin E on ovarian follicles against increased oxidative stress following diazinon application. Those authors showed that diazinon reduced proliferation in secondary and Graff follicles, while vitamin E significantly increased proliferative cells in secondary and Graff follicles (Sargazi et al. 2019). Examination of the ovarian volume showed higher antral follicle, corpus luteum, and cortex volumes in the Hypert + Vit E group compared with the Hypert group. It may therefore be suggested that vitamin E exerts a protective effect against the deleterious effects of hyperthyroidism on ovarian tissue. The results obtained using the optical fractionator method also indicated that vitamin E exhibits a protective effect against the adverse effects of hyperthyroidism on ovarian follicle numbers, although that protective effect did not reach statistical significance. The lack of significance in the numerical data may be attributable to the insufficient nature of the daily dose and application period of vitamin E applied against the oxidative stress caused by hyperthyroidism.

The decreased cortex volume and increased connective tissue volume in the Hypert group ovarian tissues are similar to the pathological features of ovarian fibrosis. Studies have shown that excessive increases in TGF-β1 and ROS levels are among the main causes of ovarian fibrosis (Wang et al. 2020). Vitamin E may therefore prevent ovarian fibrosis by suppressing excessively increased tissue ROS and TGF-β1 concentrations. No previous research has associated hyperthyroidism and ovarian fibrosis, and further studies are therefore needed on the subject.

Vitamin E supplementation protected FSH levels in the groups with induced hyperthyroidism. Our results are consistent with other studies showing that vitamin E may play an important role in regulating hormone production in the human and rat pituitary–gonadal axes (Bassey et al. 2018). Selenium and vitamin E supplementation in patients with infertility with latent premature ovarian insufficiency has been shown to raise anti-Mullerian hormone levels and increase antral follicle numbers and ovarian volume in such women (Safiyeh et al. 2021). Research evaluating the effects of vitamin E supplementation on hormonal functions, and inflammatory and oxidative markers in women with polycystic ovary syndrome, observed that such supplementation improved the lipid profile and increased FSH and progesterone concentrations (Tefagh et al. 2022). The decrease in FSH levels in the Vit E group in the present research is consistent with those previous studies. Very few studies have investigated the positive effects of vitamin E on the pituitary gland through exposure to hyperthyroidism. Studies evaluating different mechanisms are now needed to clarify whether vitamin E has a direct effect on sexual hormone production or whether low FSH and LH levels are a compensatory mechanism that increases estrogen or progesterone levels.

## Conclusion

The biochemical, histopathological, and stereological analyses in this study show that vitamin E exerts a protective effect on the pituitary–ovarian axis by providing oxidant–antioxidant homeostasis against the deleterious effects of hyperthyroidism. We therefore think that this study provides useful insights concerning infertility problems caused by hyperthyroidism, and the protective effects of vitamin E.

## Data Availability

No datasets were generated or analyzed during the current study.
